# The envelope of passive motion allowed by the capsular ligaments of the hip

**DOI:** 10.1016/j.jbiomech.2015.09.002

**Published:** 2015-11-05

**Authors:** Richard J. van Arkel, Andrew A. Amis, Jonathan R.T. Jeffers

**Affiliations:** a Department of Mechanical Engineering, Imperial College London, London SW7 2AZ, Unitrd Kingdom; b Musculoskeletal Surgery, Department of Surgery and Cancer, Imperial College London, Charing Cross Hospital, London W6 8RF, United Kingdom

**Keywords:** Hip, Capsule, Ligaments, Range of motion, Slack

## Abstract

Laboratory data indicate the hip capsular ligaments prevent excessive range of motion, may protect the joint against adverse edge loading and contribute to synovial fluid replenishment at the cartilage surfaces of the joint. However, their repair after joint preserving or arthroplasty surgery is not routine. In order to restore their biomechanical function after hip surgery, the positions of the hip at which the ligaments engage together with their tensions when they engage is required. Nine cadaveric left hips without pathology were skeletonised except for the hip joint capsule and mounted in a six-degrees-of-freedom testing rig. A 5 N m torque was applied to all rotational degrees-of-freedom separately to quantify the passive restraint envelope throughout the available range of motion with the hip functionally loaded. The capsular ligaments allowed the hip to internally/externally rotate with a large range of un-resisted rotation (up to 50±10°) in mid-flexion and mid-ab/adduction but this was reduced towards the limits of flexion/extension and ab/adduction such that there was a near-zero slack region in some positions (*p*<0.014). The slack region was not symmetrical; the mid-slack point was found with internal rotation in extension and external rotation in flexion (*p*<0.001). The torsional stiffness of the capsular ligamentous restraint averaged 0.8±0.3 N m/° and was greater in positions where there were large slack regions. These data provide a target for restoration of normal capsular ligament tensions after joint preserving hip surgery. Ligament repair is technically demanding, particularly for arthroscopic procedures, but failing to restore their function may increase the risk of osteoarthritic degeneration.

## 1 Introduction

Anatomical limits to the range of motion (ROM) of the hip joint are important to prevent impingements, which can lead to serious clinical problems. For young adults, femoroacetabular impingement (FAI) in the native hip causes pain and trauma to the acetabular labrum or articular cartilage and can, in the long-term, lead to osteoarthritis (Leunig and Ganz, 2014). For total hip arthroplasty (THA) patients, impingements cause subluxations and subsequent edge loading and high wear (De Haan et al., 2008, Esposito et al., 2012) or dislocation (Bartz et al., 2000). Consequently, there is much benefit to be gained from understanding how the natural hip limits ROM to prevent impingement.

The majority of hip ROM research considers how impingement is influenced by bony hip morphology only, and the effects that surgery can have on this (Audenaert et al., 2011, Audenaert et al., 2012, Bedi et al., 2011a, Burroughs et al., 2005, D׳Lima et al., 2000, Kessler et al., 2008, Kubiak-Langer et al., 2007, Nakahara et al., 2011, Tannast et al., 2007, Tannast et al., 2012). Many of these studies investigate joint morphology or implant shape/position in isolation and find that there is a non-symmetrical range of hip rotation. In extension, the hip has a large range of internal rotation but is at risk of impingement in external rotation. Conversely, in deep flexion the hip has a large range of external rotation but is at risk of impingement in internal rotation (Burroughs et al., 2005, Kessler et al., 2008, Nakahara et al., 2011, Tannast et al., 2012). However, clinical measurements of hip rotation suggest ROM is more symmetrical than these models indicate (Boone and Azen, 1979, Roach and Miles, 1991) and indeed recent research has described how the soft tissues also limit hip ROM (Incavo et al., 2011, Safran et al., 2013). Including these tissues in ROM models has demonstrated that variations in hip geometry which affect ROM within the soft-tissue passive restraint envelope are more important than variations outside it (Incavo et al., 2011).

Of the hip soft-tissues, the influence of the hip capsular ligaments on ROM restraint is particularly important to consider because any intra-articular hip surgery necessarily involves an incision to these ligaments to gain access to the hip, whether open (Leunig and Ganz, 2014) or arthroscopic (Domb et al., 2013) joint preserving surgery, or THA (Masonis and Bourne, 2002). In vitro data indicate the capsular ligaments limit the available ROM in the native hip (Myers et al., 2011, Safran et al., 2013, van Arkel et al., 2015), and that when they pull taut in deep flexion they may protect the hip against posterior edge loading (van Arkel et al., 2015). In vitro data also demonstrate that synovial fluid flows from the central intra-articular compartment of the hip to the peripheral compartment (Dwyer et al., 2014) and it has been suggested that tightening of the capsular ligaments may circulate synovial fluid back to the central compartment again (Field and Rajakulendran, 2011). There are therefore several possible biomechanical functions of the capsular ligaments and several groups advocate their repair after joint preserving surgery (Bedi et al., 2011b, Domb et al., 2013, Frank et al., 2014). However it remains a technically demanding task and is not routinely performed (Domb et al., 2013, Ilizaliturri, 2009). This is a concern as failure to restore these biomechanical functions may increase the risk of osteoarthritis progression.

Most hip ligament research focusses on a neutral ab/adduction swing path so it remains unclear when the ligaments engage as ab/adduction varies (Martin et al., 2008, Myers et al., 2011). There is therefore a lack of baseline experimental data describing the positions throughout ROM where the capsular ligaments pull taut to restrain rotation of the native hip, and what rotational stiffness of restraint they provide when they do. These data would be useful for both assessing the importance of the capsular repair for a patient (Domb et al., 2013) as well as performing the repair to restore natural biomechanics.

The aims of this study were to quantify the ligamentous passive restraint envelope for the hip when it is functionally loaded throughout the whole ROM, and to quantify the amount of rotational stiffness provided by the capsular ligaments once taut. This would provide the surgeon with an objective target to restore ligament biomechanics following early intervention surgery. The null hypothesis is that the passive rotation restraint envelope does not vary throughout hip ROM.

## 2 Materials and methods

### 2.1 Specimen preparation

**Fig. 1:**
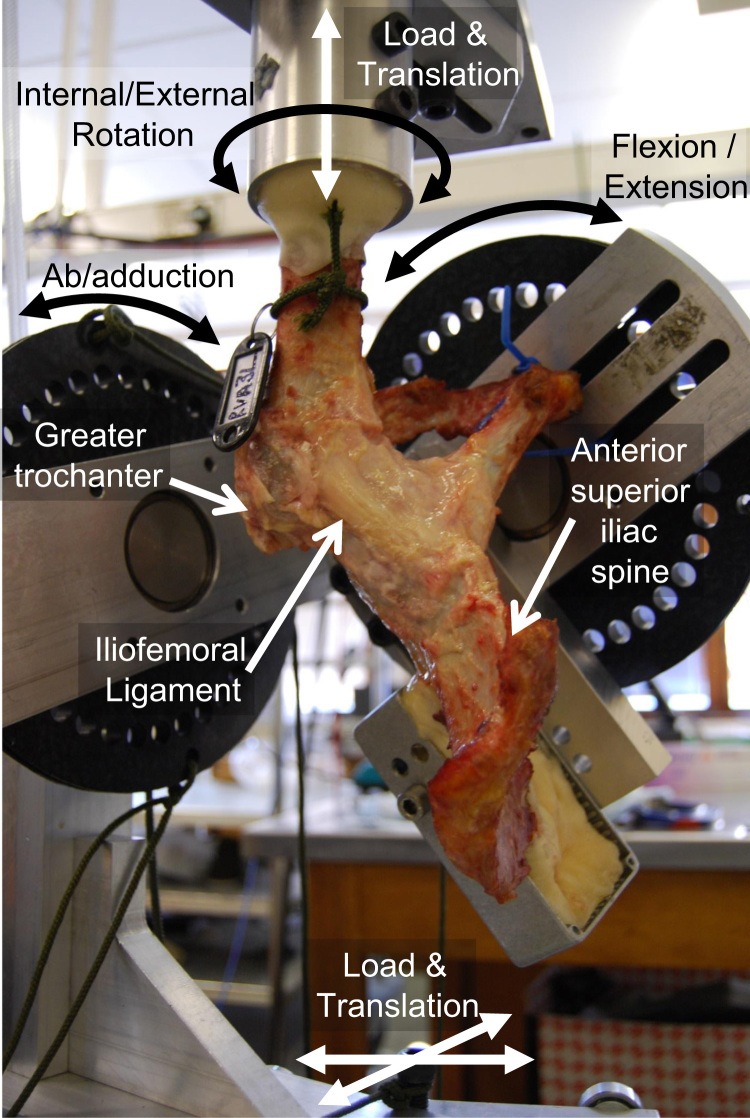
Anterolateral view of a hip in the testing rig in flexion, adduction and external rotation. In this photograph, the iliofemoral capsular ligament can be seen to be taut and resisting a 5 N m external rotation torque being applied by the servo-hydraulic machine. Internal/external rotation and vertical loads are controlled by a dual-axis servo-hydraulic machine (not shown) and horizontal loads and translations are applied using dead weights and a low-friction *x*–*z* table (not shown).

Following approval from the local Research Ethics Committee, 10 fresh-frozen cadaveric pelvises (six male) with full length femurs were defrosted and skeletonised, carefully preserving the hip joint capsule. Guide holes were drilled into the left posterior superior iliac spines and femoral shaft before bisecting the pelvis and transecting the femoral mid-shaft. The guide holes based on the contra-lateral pelvis and femoral epicondyles were then used to mount the hip into a six-degrees-of-freedom testing rig (Fig. 1) according to the International Society of Biomechanics (ISB) coordinate system (Wu et al., 2002). Neutral flexion, rotation and ab/adduction equated to a standing upright position (when the ISB pelvic *X*–*Y*–*Z* axes aligned with the femoral *x*–*y*–*z* axes).

### 2.2 The testing rig

The rig comprised of a femoral-fixture that was attached to a dual-axis servo-hydraulic materials-testing-machine (model 8874, Instron Ltd, High Wycombe, United Kingdom) equipped with a two-degree-of-freedom (torsion/tension) load cell and a pelvic-fixture that could constrain, release or load the other four-degrees-of-freedom (Fig. 1). Pure moments could be applied in all three physiological directions: internal/external rotation torque through the rotating axis of the servo-hydraulic machine and flexion/extension or ab/adduction torques with a pulley and hanging-weights couple. This meant that any hip could freely rotate about its natural centre, unconstrained, without affecting the magnitude of applied torque. Fixed angular positions could be applied using position control on the servo-hydraulic machine or with screw clamps on the pulleys. Femoral proximo-distal loads (along the femoral *y*-axis) were applied by operating the vertical axis of the servo-hydraulic machine in load control whilst an *x*–*z* bearing table and a hanging weight applied joint reaction force components in the transverse plane; translations in the secondary translational degrees-of-freedom *x*–*y*–*z* were free to occur in response to the applied load and ligament tension.

### 2.3 Testing protocol

For each specimen, all tests were performed at room temperature on the same day without removing the specimen from the testing rig. The specimens were kept moist using regular water spray. With the femur in the neutral position, a fixed compressive load in the coronal plane of 110 N angled 20° medially/proximally relative to the mechanical axis of the femur was applied. This loading vector was held constant relative to the femur whilst the pelvis was flexed/extended and ab/adducted to apply ROM. As load direction was relative to the femur this meant that, for example, if the hip was flexed to 90° (in the rig this would mean the pelvis was rotated 90°) then the load would be applied in the transverse plane. This loading direction was chosen based on the mean (±S.D.) direction of the hip contact force relative to the femur during functional tasks reported in HIP98 (Bergmann et al., 2001): 18±5° medially/proximally and 0±6° anteriorly/proximally for an average patient walking fast/slow, up/down stairs, standing up, sitting down, and knee bend.

For each specimen, the ROM with the joint capsule intact was established by applying 5 N m extension/flexion torques with the hip joint in neutral rotation and ab/adduction to define a value of extension (EXT) and deep flexion (FLX) for the hip. Then, with the joint still in neutral rotation, 5 N m ab/adduction torques were applied to measure values of high abduction (ABD) and high adduction (ADD) at six different flexion angles (EXT, F0°, F30°, F60°, F90° and FLX). Finally, 5 N m torques were applied in internal/external rotation at 30 different hip positions; all possible combinations of ABD, AB20° (abducted to 20°), A0° AD20° (adducted to 20°) and ADD at all six flexion/extension angles. At each hip position, these rotation movements were applied by the servo-hydraulic machine using a sinusoidal waveform (neutral→external→neutral→internal→neutral) with a 10 s period whilst continuously recording the angle of rotation and passive rotation resistance. Each movement was performed twice and data were analysed from the second iteration.

In order to assess specimen morphology, following testing, the following measurements were made: femoral head diameter, offset, anteversion, neck-shaft angle and head/neck ratio (Doherty et al., 2008), as well as acetabular centre edge angle and depth ratio (Cooperman et al., 1983). The *α* and *β* angles, and the anterior neck offset ratio were also measured (Meyer et al., 2006). Specimens with *α*>55° or centre-edge angle <25° were considered abnormal and were excluded from the data analyses (Ellis et al., 2011, Jacobsen, 2006).

### 2.4 Data analysis

Internal/external torque–rotation curves for each specimen in each hip position were plotted using MatLab (version 2011b, The MathWorks, Inc., Austin, TX). The angular positions where the hip joint motion transitioned from slack to stiff were identified by finding the first points where the torque–rotation gradient exceeded 0.03 N m/° for both internal and external rotation. This value of 0.03 N m/° was determined from pilot data by visually inspecting plots of the torque–rotation data alongside the calculated gradient values.

The slack/stiff transition points were then used to calculate three parameters for further analysis: the range of un-resisted rotation (slack region), the location of the mid-slack point and the change in rotation from the transition point to 5 N m of passive rotation restraint (slack-to-taut). In cases where there was continually passive restraint with no slack region, the mid-slack angle was defined at 0 N m passive resistance torque (the *x*-intercept). Finally, the gradient values were additionally used to quantify the aggregate torsional stiffness provided by the capsular ligaments at the point of 5 N m passive resistance.

### 2.5 Statistical analysis

The values recorded at AD20° and AB20° could not be included in the repeated measures analyses because not all hips could reach these positions in extension or deep flexion. Data were analysed in SPSS (version 22, SPSS Inc, Chicago, Illinois) with two- or three-way repeated measured analyses of variance (RMANOVA). The independent variables were the angles of hip flexion (EXT, F0°, F30°, F60°, F90° and FLX) and hip ab/adduction (ABD, A0° and ADD) for the two-way analyses, with an additional factor of direction of rotation (ER and IR) for the three-way analyses. Four dependant variables were analysed: the range of un-resisted rotation (two-way analysis), the angle of mid-slack (two-way analysis), the angular change from the transition point to 5 N m passive restraint (three-way analysis) and finally the torsional stiffness of the hip at the point of 5 N m restraint (three-way analysis). Post-hoc paired *t*-tests with Bonferroni correction were applied when differences across tests were found. The significance level was set at *p*<0.05. The number of post-hoc comparisons at a given level of flexion was different from that at a given level of ab/adduction. Therefore adjusted *p*-values, multiplied by the appropriate Bonferroni correction factor in SPSS, have been reported rather than reducing the significance level.

## 3 Results

One male hip had a visibly aspherical head (*α*=64°) and was excluded from the data analysis. External rotation data for one female specimen was lost due to the capsule rupturing from the bone when 5 N m torque was applied in external rotation meaning that subsequent hip rotation results are presented for only eight specimens. Morphological measurements of these specimens are presented in Table 1.

**Fig. 2:**
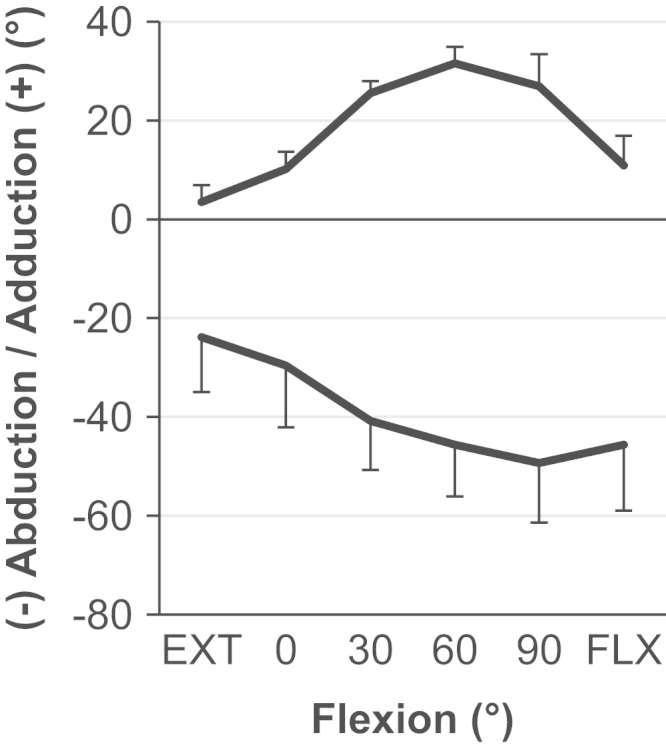
The mean ab/adduction with standard deviation (*n*=9) when 5 N m torque was applied as flexion was varied whilst internal/external rotation was fixed in the neutral position.

Under 5 N m torque the mean (± standard deviation) hip joint flexion was 112±10° and extension was −12±7°. The range of hip joint ab/adduction varied with the angle of hip flexion; it was largest in 60–90° of flexion and smallest in hip extension (Fig. 2).

### 3.1 The passive restraint envelope: the range of un-resisted rotation

**Fig. 3:**
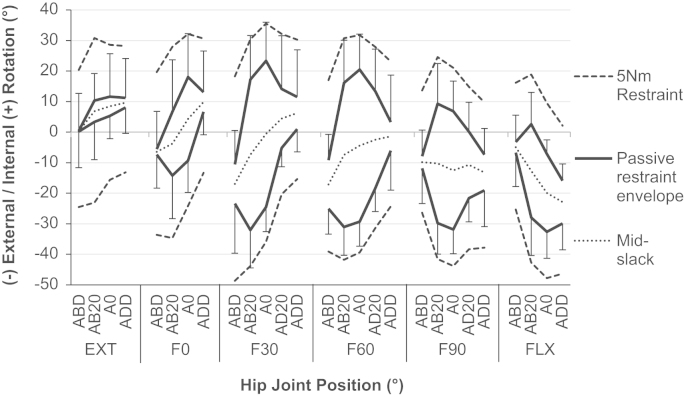
The rotation passive restraint envelope (with standard deviation, *n*=8), the points of mid-slack and the 5 N m measurement boundaries across a complete hip range of motion. It can be seen that there was a greater range of un-resisted rotation (space between the solid black lines) in mid-flexion and mid-ab/adduction than when the hip was deeply flexed/extended, or highly ab/adducted. It can also be seen that the hip was more open to internal rotation in extension, and external rotation in flexion as the mid-slack points (grey dots) shifted to external rotation as hip flexion was increased. However once the ligaments had started to restrain hip rotation, the internal/extenral rotation restraint is more symmetrical (equal spacing between solid black lines and dashed grey lines at each position).

**Fig. 4:**
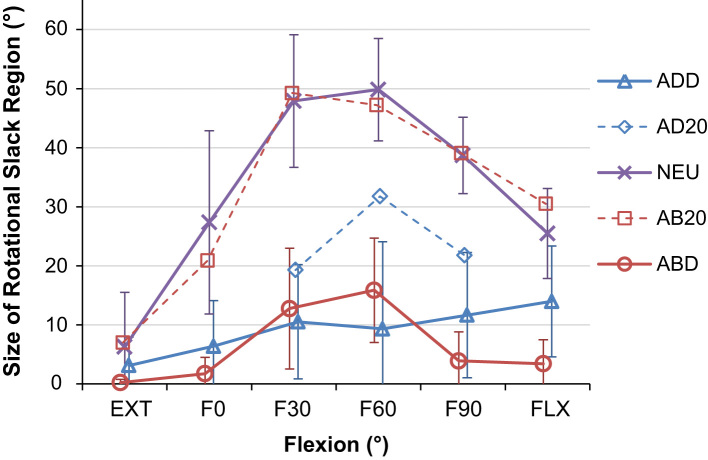
The mean range of rotational slack as a function of flexion for different ab/adduction values (with 95% confidence intervals). It can be seen that the hip tightens in deep flexion and extension, and high abduction/adduction whilst the greatest range of slack occurs when the hip is neutrally or partially abducted and mid-flexed.

The range of un-resisted rotation (slack region) varied with both the angle of hip flexion and ab/adduction (Fig. 3) and the effect of flexion on the slack region was found to be dependant on the level of ab/adduction and vice-versa (*p*<0.001). The post-hoc analysis showed that the slack region in neutral ab/adduction was greater than that in high ab/adduction (all *p*<0.014, Table 2 and Fig. 4). The largest difference was at F60° where the mean slack region was 41±13° larger in neutral ab/adduction than when the hip was highly adducted (*p*<0.001). Similarly, the hip had a greater slack region in mid-flexion compared to extension and deep flexion (with neutral abduction, all *p*<0.006, Table 2 and Fig. 4). The largest difference was at F60° where the mean slack region was 44±15° larger at F60° than EXT (*p*=0.001).

### 3.2 The passive restraint envelope: mid-slack

**Fig. 5:**
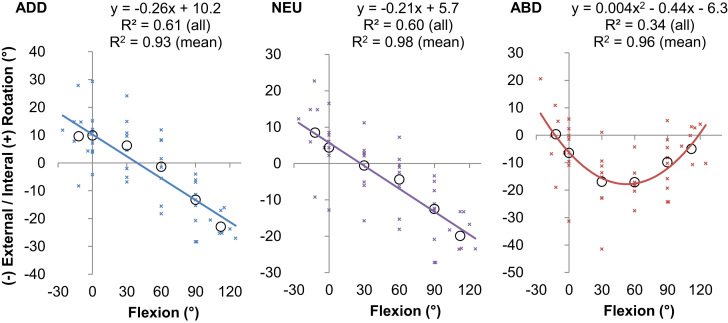
The position of the mid-slack point as a function of flexion for (a) full adduction, (b) neutral adduction and (c) full abduction. When the hip is highly adducted (left) or neutrally ab/adducted (middle) then the range of un-resisted hip rotation is biased to internal rotation in extension and external rotation in flexion as demonstrated by the linear transition of the mid-slack point with increasing hip flexion. When the hip is highly abducted (right) then the mid-slack point positioned most in external rotation in mid-flexion and is better described by a second order polynomial. The equations provided represent a good model for the raw data from all specimens (small crosses, all *R*^2^≥0.34) and an excellent model for an average hip as demonstrated by the mean data at each flexion angle (black circles, all *R*^2^≥0.93).

The position of the mid-slack point also varied with the angle of hip flexion and abduction (Fig. 3) with an interaction effect between flexion and ab/adduction (*p*<0.001). Post-hoc analyses showed that, for both neutral and high adduction, the mid-slack point was found with the hip internally rotated in extension, externally rotated in deep hip flexion (*p*<0.001, Fig. 5a and b). However, when the hip was highly abducted, no difference was detected between the position of the mid-slack point in deep flexion and extension. Instead, the mid-slack point was found with the hip externally rotated in mid flexion, resulting in a parabolic-like shift in the location of the mid-slack point (*p*<0.028 for both F30° and F60° compared to extension, Fig. 5c).

### 3.3 Slack-to-taut and torsional stiffness

**Fig. 6:**
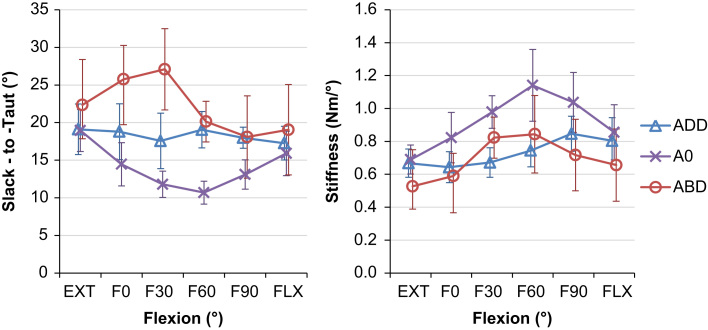
Mean angular change between the transition point and 5 N m of rotational restraint, left, and torsional stiffness, right as a function of flexion for different ab/adduction values (with 95% confidence intervals). It can be seen that for neutral ab/adduction, as slack-to-taut decreases, stiffness increases and vice-versa.

Neither the angular change from the transition point to 5 N m passive restraint (slack-to-taut) nor the torsional stiffness at 5 N m restraint was affected by a three-way interaction between flexion, ab/adduction and rotation direction. However, both dependant variables did vary with hip position with a two-way interaction detected between flexion and ab/adduction across both directions of rotation (for slack-to-taut *p*=0.006*,* and for torsional stiffness *p*=0.036). Post-hoc analysis detected differences in similar positions to those found for the slack region (in mid-flexion and mid-ab/adduction, Fig. 3). Generally, when the slack region increased, torsional stiffness increased and slack-to-taut decreased (Table 2 and Fig. 6).

## 4 Discussion

The most important finding of this study was that the passive restraint envelope for hip rotation varied with the angle of flexion/extension and ab/adduction (Fig. 3). In a position of mid-flexion and mid-ab/adduction there were large slack regions where the capsular ligaments provided no rotational restraint (Fig. 4), which indicate a large in-vivo ROM that allows the hip to move freely under the action of hip muscles during many daily activities. Conversely, towards the extremes of hip ROM (in positions of deep flexion/extension or high levels of ab/adduction) there was a minimal/non-existent slack region, thus limiting the available range of rotation in positions where the hip is vulnerable to impingement and/or subluxation. The results also showed that internal/external rotation restraint is not symmetrical; the mid-slack point displayed a shift from an internally rotated position in extension to an externally rotated position in hip flexion (Fig. 5).

Our results do not distinguish between capsular rotational restraint and that from labral impingement, but provide an aggregate rotational restraint from the peri-articular tissues. However, within the 5 N m restraint boundaries examined, our previous research found that the mean labral contribution to rotational restraint only exceeded 20% in 6/36 hip positions and was less than the capsular contribution to rotational restraint in all hip positions (*p*<0.05) (van Arkel et al., 2015). These labral impingements were observed most frequently when the hip was in high abduction, which may be the cause of the parabolic shift of the mid-slack point in high abduction (Fig. 5), and also the few hip positions in low flexion and high abduction where slack-to-taut and torsional stiffness seemingly both increase (Fig. 6). Another limitation was the high mean age of the cadaveric specimens; they are better matched to patients undergoing THA than those receiving early intervention surgery. Our study also did not consider the effects of osteoarthritis on capsular stiffness, or how a smaller head size for a THA may affect the ability of the capsule to wrap around the head and tauten (Colbrunn et al., 2013). By only including normal hips in the study it was also not possible to address whether hips suffering from FAI have normal capsular anatomy/function. However, studies have suggested similarities between hip capsule dimensions in pathological hips (Weidner et al., 2012) and normal hips (Stewart et al., 2002).

**Fig. 7:**
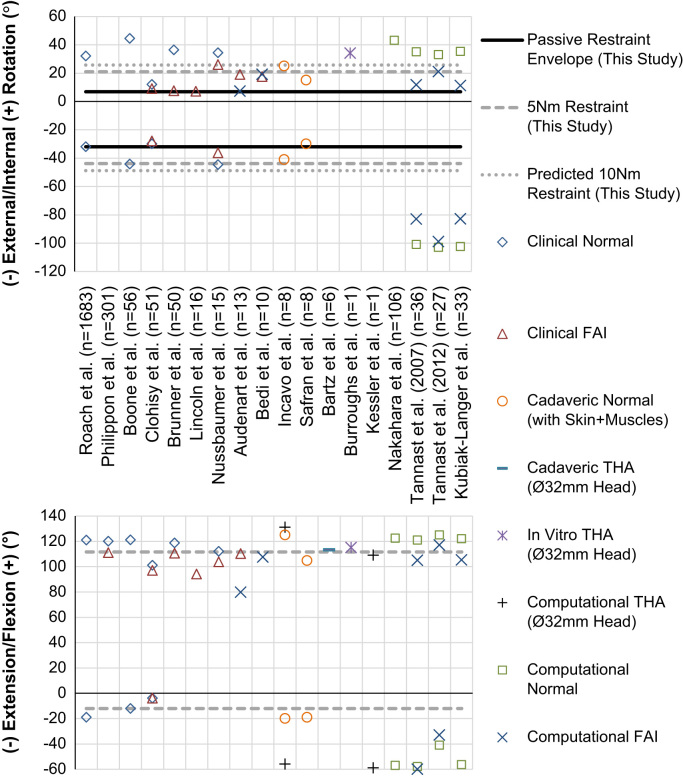
A comparison between clinical, experimental and computational range of motion measurements and the results from the present study for internal and external rotation at 90° flexion with neutral ab/adduction (top), and for flexion/extension (bottom). It can be seen that the passive restraint envelope (for un-resisted rotation) measured in the present study was within clinical measurements for normal subjects, compares well to previous cadaveric work, and was always less than results from studies which only considered bony impingement as a limit to hip rotation (computational studies). The predicted 10 N m restraint values were calculated using the mean torsional stiffness measured at 5 N m restraint.

In Fig. 7, the passive restraint envelope measured in this study is overlaid on ROM data taken from 18 studies with a total of more than 2400 subjects, which include clinical goniometer readings, in-vitro experiments including skin and muscles and computational impingement models. Our data are in good agreement with other cadaver based studies, but the passive restraint envelope is typically less than clinical measurements. This is to be expected as clinical ROM measurements usually measure the relative movement between the thigh and trunk, thus including contributions from the lumbar spine, sacro-iliac joint as well as the anatomic hip joint. However, the ROM measured in the current study was always less than that measured when only bone-on-bone impingement was considered for normal hips (the computational studies in Fig. 7) indicating the capsular ligaments engage to prevent impingement. The impingement-free range of rotation measured in bone–bone impingement studies is biased towards internal rotation in extension (Kessler et al., 2008, Tannast et al., 2012), and external rotation in flexion (Kubiak-Langer et al., 2007, Tannast et al., 2012). Our data indicate capsular rotation restraint guides the available range of rotation towards these impingement-free positions as the mid slack point shifts 30° from an internally rotated position in extension to a more externally rotated position in deep flexion (Fig. 5).

Several authors have reported the total resistance to hip joint distraction/dislocation (Elkins et al., 2011, Ito et al., 2009), the stiffness of individual ligaments (Hewitt et al., 2002), their contribution to hip rotation restraint (Myers et al., 2011, Smith et al., 2014, van Arkel et al., 2015), or their influence on hip ROM (Safran et al., 2013). However to our knowledge there are no studies measuring the slack region, or the angular change required to tauten the ligaments or torsional stiffness provided by an intact capsule once the ligaments are taut. This study quantifies these variables and the findings correlate well with the understanding of the anatomy of the capsular ligaments. The four capsular ligaments available for limiting hip rotation (medial and lateral arms of the iliofemoral, ischiofemoral and pubofemoral) are the same ligaments which can generate resistive moments against deep flexion/extension or high ab/adduction (Fuss and Bacher, 1991, Martin et al., 2008). This explains the reduced hip rotation slack region observed in the more extreme hip positions (Fig. 3, Fig. 4) as the ligaments are recruited to limit both large movements of the lower limb (flexion/extension or ab/adduction) and hip rotation. It also explains the reduced rotational stiffness (Fig. 6) in these hip positions as the ligament fibres do not align to purely resist hip rotation but also the other movements. Conversely in mid-flexion and mid-ab/adduction, there is a large slack region available as the ligaments are not resisting movements in any direction. When the hip is excessively rotated in these mid-ROM positions such that the ligaments start to tauten, the ligaments develop high levels of torsional stiffness in small angular changes (Fig. 6) as the fibres are orientated more perpendicularly to the axis of hip rotation, directly opposing the movement.

In conclusion, to our knowledge, this is the first study to quantify the hip positions where the capsular ligaments restrain hip rotation and those where the joint is slack, how much rotation is required to tighten the ligaments, and how much rotational stiffness is provided by them once taut. These results provide a benchmark for the normal joint that can be used as a target for capsular repair in joint preserving surgery, and enable the restoration of capsular biomechanical function after surgery.

## Conflicts of interest

None.

## Acknowledgements

This study was funded by the Wellcome Trust and EPSRC [088844/Z/09/Z] and the Institution of Mechanical Engineers. The dual-axis Instron materials-testing-machine was provided by an equipment grant from Arthritis Research UK.

## Figures and Tables

**Table 1:** Morphological measurements of the eight hips included in the data analysis.

**Measurement**	**Mean±S.D.**
Age	76±9
Femoral head diameter (mm)	50±5
Femoral anteversion (deg)	9±11
Femoral neck-shaft angle (deg)	130±4
Femoral offset (mm)	36±9
Femoral head/neck ratio	1.40±0.07
Femoral anterior neck offset ratio	0.18±0.02
Femoral alpha angle (deg)	48±6
Femoral beta angle (deg)	45±5
Acetabular centre edge angle (deg)	41±9
Acetabular depth ratio	274±22

**Table 2:** All significant increases/decreases measured for the slack region, slack-to-taut and torsional stiffness. Hip positions where there were common significant differences (i.e. the key findings of the study) are in italics.

**Hip position**	**Significant differences (with *p*-values)**					
**Slack region**		**Slack-to-taut**		**Torsional stiffness**	
**ABD**	None	–	None	–	F30>EXT	0.022
F60>F90	0.018
**A0**	*F30>EXT*	0.002				
*F60>EXT*	0.001	*F30<EXT*	0.044		
*F90>EXT*	0.005	*F60<EXT*	0.003	*F30>EXT*	<0.001
*F60>F90*	0.006	*F90<EXT*	0.040	*F60>EXT*	0.004
F60>FLX	0.008	*F60<F90*	0.046	*F90>EXT*	0.012
F90>FLX	0.022				
**ADD**	None	–	None	–	F90>F0	0.001
F90>F30	0.033
**EXT**	None	–	*A0<ABD*	0.050	*A0>ABD*	0.043
**F0**	*A0>ABD*	0.013	*A0<ABD*	0.010	*A0>ADD*	0.048
*A0>ADD*	0.012	ADD<ABD	0.035
**F30**	*A0>ABD*	0.001	*A0<ABD*	0.001	ABD>ADD	0.031
*A0>ADD*	<0.001	*A0<ADD*	0.023	*A0>ADD*	<0.001
ADD<ABD	0.022
**F60**	*A0>ABD*	<0.001	*A0<ABD*	<0.001	*A0>ABD*	0.008
*A0>ADD*	<0.001	*A0<ADD*	<0.001	*A0>ADD*	0.028
**F90**	*A0>ABD*	<0.001	*A0<ADD*	0.006	*A0>ABD*	0.003
*A0>ADD*	0.001
**FLX**	A0>ABD	0.001	None	–	None	–
A0>ADD	0.008
